# Codelivery of Genistein and miRNA-29b to A549 Cells Using Aptamer-Hybrid Nanoparticle Bioconjugates

**DOI:** 10.3390/nano9071052

**Published:** 2019-07-23

**Authors:** Koita Sacko, Karthik Thangavel, Sunday A. Shoyele

**Affiliations:** Department of Pharmaceutical Science, College of Pharmacy, Thomas Jefferson University, Philadelphia, PA 19107, USA

**Keywords:** hybrid nanoparticles, genistein, microRNA-29b, combination therapy, mucin-1 MUC1, aptamer, non-small cell lung cancer

## Abstract

This study aimed to evaluate the anti-cancer effect of a combination therapy of miRNA-29b and genistein loaded in mucin-1 (MUC 1)-aptamer functionalized hybrid nanoparticles in non-small cell lung cancer (NSCLC) A549 cell line. Genistein-miRNA-29b-loaded hybrid nanoparticles (GMLHN) was prepared and characterized. Particle size and zeta potential were measured using photon correlation spectroscopy (PCS). Encapsulation efficiency and loading efficiency were determined using HPLC. Preferential internalization of MUC 1-aptamer functionalized GMLHN by A549 cells was evaluated and compared to normal MRC-5 cells. The ability of GMLHN to downregulate targeted oncoproteins Phosphorylated protein kinase, strain AK, Thymoma (Phosphorylated protein kinase B) (pAKT), Phosphorylated phosphoinositide 3-kinase (p-PI3K), DNA (cytosine-5-)-methyltransferase 3 beta (DNMT3B) and Myeloid Cell Leukemia Sequence 1 (MCL 1) was evaluated using western blot, while antiproliferative effect and ability to initiate apoptosis was also assessed in A549 cells. MUC 1-aptamer functionalized GMLHN nanoparticles were prepared. These nanoparticles were preferentially internalized by A549 cells but less so, in MRC-5 cells. pAKT, p-PI3K, DNMT3B and MCL 1 were efficiently downregulated by these nanoparticles without affecting the levels of AKT and PI3K in A549 cells. GMLHN demonstrated a superior antiproliferative effect compared to individual genistein and miRNA-29b-loaded nanoparticles. Results generated were able to demonstrate that genistein-miRNA-29b-loaded hybrid nanoparticles (GMLHN) could be a potential treatment modality for NSCLC because of the ability of the payloads to attack multiple targets.

## 1. Introduction

The leading cause of cancer death is non-small cell lung cancer (NSCLC). In the United states, only a five-year survival rate of 15% has been achieved for all stages of NSCLC [1,2]. Recent breakthroughs in the development of chemotherapy has not reflected significantly in cure rates, which still stands at less than 18% [3]. Due to the fact that cytoreductive surgery has not been very effective because of the quick spread of NSCLC, chemotherapy in combination with radiotherapy has been the treatments of choice. However, resistance of cancer cells to chemotherapy has led to limited efficacy of conventional chemotherapy [3,4,5]. The investment of several billions of dollars in research on cancer drug development has not had any substantial decrease in mortality for several decades [6]. Hence, there is a need to try different modalities, to help improve the outcome of chemotherapy in NSCLC.

Our present study aims to co-encapsulate miRNA-29b with genistein in hybrid nanoparticles (GMLHN) to produce a combination therapy that will effectively treat NSCLC. Genistein is naturally found in soy products and is known to elevate the antitumor effect of some chemotherapeutic agents in several tumors [7]. Previously, genistein is known to inhibit the growth of lung cancer by downregulating essential oncoproteins, phosphorylated protein kinase, strain AK, Thymoma (pAKT), and phosphorylated phosphoinositide-3 kinase (p-PI3K) [7].

Specifically, genistein was shown to inhibit A549 cells proliferation via miR-27a-mediated MET signaling [8]. Genistein has also been shown to have in vitro anticancer effect in breast cancer and prostate cancer cells [8]. Considering the fact that these genistein and miRNA-29b attack different targets in cancer, we hypothesize that the combination of miRNA-29b and genistein will provide an additive effect in the treatment of NSCLC. We believe that the simultaneous induction of cell death by an anticancer drug (genistein) and suppression of antiapoptotic defense/stimulation of apoptosis (miRNA-29b) will enhance the treatment of NSCLC. To enhance selective delivery of these payloads to NSCLC, Genistein-miRNA-29b-loaded hybrid nanoparticles (GMLHN) are conjugated to MUC 1-aptamer for targeted delivery to NSCLC while avoiding all the well-documented side effects associated with miRNA-nanoparticle therapeutics. MUC 1 is a cell membrane-located oncoproteins. Its level of expression in NSCLC is abnormally high in comparison to normal cells [9,10]. Immunohistochemistry analysis has shown that it is abnormally high in 80% of adenocarcinoma cells of the lung [9,10]. Taking advantage of this abnormally high expression in NSCLC, we have conjugated MUC 1 aptamer to the surface of our hybrid nanoparticles to achieve an optimized targeting of miRNA-29b and genistein to NSCLC [11,12].

## 2. Materials and Methods

### 2.1. Materials

Lyophilized human immunoglobulin G was obtained from Equitech-Bio Inc. (Kerrville, TX, USA). Fetal bovine serum (FBS), Poloxamer-188, as well as 4,6-diamidino-2-phenylindole (DAPI) were purchased from Thermo Fisher Scientific, Waltham, MA, USA. GE Healthcare Bio-Sciences Corp., Piscataway, NJ, USA supplied Aptamer against anti-MUC1 aptamer. The design tool on the website of GE Healthcare was used to attach a C12 spacer to the 3′-end of 5′-GCA GTT GAT CCT TTG GAT ACC CTG G-C12H25-NH_2_-3′. One variant of the anti-MUC1 aptamer was made fluorescent by attaching FITC to the 5′ position. The sequence for the MUC 1-aptamer was obtained from Wang et al. [10]. 6-FAM-labeled-siGLO used as a fluorescent-control miRNA, was also obtained from GE Healthcare Bio-Sciences Corp. Hydrochloric acid solution was supplied by Thermo Fisher Scientific. All other primary antibodies were purchased from Cell Signalling (Danvers, MA, USA). Pierce Radioimmunoprecipitation assay (RIPA) lysis buffer was bought from Thermo Fisher Scientific. miRIDIAN mimic negative control and miRIDIAN mimic miR-29b were obtained from GE Healthcare Bio-Sciences Corp. Genistein was obtained from Shaanxi Yi, Company Limited (Shaanxi, China).

### 2.2. Cell Culture

A549, that was originally obtained from a 14-week male Caucasian fetus and MRC-5 cell line were obtained from American Type Culture Collection, Rockville, MD, USA. A549 and MRC-5 cells were maintained in F12 K medium and Eagle’s Minimum Essential Medium respectively. 10% FBS was added to each media as supplements. Cells were incubated in a humidified air atmosphere with 5% carbon dioxide.

### 2.3. Methods

#### 2.3.1. Production of Nanoparticles

The nanoparticle preparation medium contained 1 mg/mL of human IgG in 0.01 N hydrochloric acid. 4.38 mg of genistein and 0.73 mg of miRNA-29b or siGLO were dissolved in the medium while gently stirred by a magnetic stirrer. While monitoring the pH of the medium, 0.01 N sodium hydroxide was slowly added to the medium until a pH value of 7 was attained. This led to the spontaneous formation of the nanoparticles. The nanoparticle suspension was allowed to continue to mix for 10 min, after which it was aliquoted into several Eppendorf tubes to be centrifuged at 2000 rpm for 5 min. Supernatants recovered were used to determine both encapsulation efficiency (EE) and loading capacity (LC), using HPLC for analysis. Nanoparticles were coating with 0.2% *v*/*v* carbonyl-functionalized poloxamer-188. Conjugation of aptamer to nanoparticles was accomplished using a published conjugation processed [10,11]. In summary, 50 μL of nanoparticle suspension (10 μg/mL in DNase, RNase-free water) was added to 100 μL of 40 mM 1-ethyl-3-(3-dimethylaminopropyl)carbodiimide hydrochloride (EDC) and 100 μL of 10 mM N-hydroxysulfosuccinimide (NHS) for 15 min at 20 °C on a magnetic stirrer. 50 μL of 1 μg/mL MUC 1-aptamer in DNase, prepared in RNAse-free water was mixed with the nanoparticles on a magnetic stirrer for 2 h at 20 °C. Aptamer-functionalized nanoparticles were separated from unconjugated aptamer, EDC and NHS by centrifuging using 30 kDa cutoff centrifugal ultrafilters (EMD Millipore, Billerica, MA, USA) at 4,000 rpm and 10 °C for 5 min. To coat the nanoparticles with poloxamer-188, aptamer-functionalized nanoparticles were then suspended in 0.2% *v*/*v* poloxamer-188, on a magnetic stirrer for 10 min.

#### 2.3.2. Particle Size Measurement

Dynamic light scattering (ZetaSizer Nano ZS; Malvern Instruments, Malvern, UK) was used to determine the particle size and the ζ (zeta potential) of produced nanoparticles. In summary, nanoparticles were suspended in water and sonicated for ~5 min in order to disperse them. A scattering angle (θ) of 173° was used to measure intensity autocorrelation. Each sample was measured three times and recorded using the Malvern Instruments software.

#### 2.3.3. Fourier Transform-infrared Spectroscopy (FT-IR)

Samples were analyzed for FT-IR using an iS10 FT-IR spectrometer (Thermo Fisher Scientific) installed with the attenuated total reflectance (ATR) crystal of a single-reflection-ATR with a diamond internal reflection crystal. Multiple spectra were obtained from different samples of nanoparticles. Background spectra were first collected before samples were mounted. Using OMNIC software, spectra for each sample were collected at 4 cm^−1^ resolution

#### 2.3.4. Scanning Electron Microscopy (SEM)

GMLHN nanoparticles suspended in double distilled deionized water were sampled on to a double–sided carbon disc on an aluminum stub. The samples were air-dried at 25 °C. Palladium was used as a coating agent before mounting the samples on a Zeiss Supra 50 V system (Carl Zeiss Meditec AG, Jena, Germany).

#### 2.3.5. Drug Release Study

Drug release from GMLHN was monitored using relevant buffers at pH values 5, 6.6, and 7.4. GMLHN (3 mg) was dispersed in 0.5 mL of relevant solution and then transferred into a dialysis membrane made of cellulose. These tubular membranes were secured at both ends. These membranes were then placed in beakers filled with 5 mL buffered solution and stored at 37 °C. The cumulative release of drug was monitored by using HPLC analysis and plotted against time

#### 2.3.6. Fluorescence Microscopy

Using an eight-well coated glass slide purchased from Discovery Labware (Tewksbury, MA), 2 × 10^4^ cell were incubated for 48 h. Media were then decanted from the wells and replaced with PBS to wash three times. Opti-Mem medium containing 100 μg/mL of nanoparticles was then added to the wells and incubated for 4 h.

After washing with PBS, 2% paraformaldehyde was used as a fixing agent, while 5% BSA was used as a blocking agent. Lysotracker-Red and DAPI were added to the cells to visualize endosomes and nucleus, respectively. Cells were then mounted overnight before collecting fluorescent images using Leica DMI 6000B microscope (Leica Microsystems, Wetzlar, Germany).

#### 2.3.7. Cell Viability Study

Each well of a 96-well plate was seeded with 1 × 10^4^ A549 cells. Following incubation for 24 h at 37 °C and 5% carbon dioxide, different concentrations of GMLHN, miRNA-29b-loaded nanoparticle, genistein-loaded nanoparticles, control miRNA-loaded nanoparticles, control miRNA-genistein-loaded nanoparticles, physical mixture of genistein and miRNA-29b, lipofectamine-miRNA-29b and PBS in Opti-MEM, were added after PBS wash. The cells were then incubated for 7 h. The samples were then replaced with F12 K medium supplemented with 10% FBS and 1% antibiotics and incubated for 3 days. Ten μL of 12 mmol/L 3-(4,5-dimethylthiazol-2-yl)-2,5-diphenyltetrazolium bromide (MTT) reagent was then added to the wells to replace the media and incubated for 4 h at 37 °C. After the MTT reagent was aspired, sterile DMSO was added and mixed thoroughly. UV absorptions of 540 nm and 650 nm were recorded.

#### 2.3.8. Western Blot Analysis

1 × 10^6^ A549 cells were seeded per well and incubated for 48 h. Cells were then treated with formulations containing genistein and miRNA-29b at a concentration of 50 nM of miRNA-29 and 1.6 M of genistein in Opti-MEM medium for 72 h. RIPA buffer containing Pierce™ Protease Inhibitor Mini Tablets were added for cell lysis and kept on ice for 30 min. Cells were then subjected to centrifugation at 10,000× g for 10 min. Following the determination of protein concentration. Proteins were subjected to electrophoresis before transferring content of the gel to nitrocellulose membrane with a pore size of 0.22 μm (Thermo Fisher Scientific). Appropriate primary antibodies overnight were added at 4 °C. After washing membranes with wash buffer thrice, secondary antibodies, conjugated with horseradish peroxidase at a dilution of 1:1,000 was added to the membrane. Pierce ECL Western chemiluminescent substrate was used to detect immune complexes.

#### 2.3.9. Cell-Death Detection ELISA

Cell death by apoptosis was measured using cell-death detection ELISA (Hoffman-La Roche Ltd., Basel, Switzerland). After seeding A549 at a density of 2 × 104/well, cells were treated with various formulations containing equivalent of 50 nM of miRNA-29 and 1.6 M of genistein in Opti-MEM medium for 72 h. Cells were lysed in incubation buffer at room temperature. The cells were then centrifuged at 4,000× g for 10 min, before collecting the supernatants. Coated plastic wells were filled with 100 μL of homogenate and incubated at 20 °C for 90 min. Wells were washed thrice after decanting the homogenate before adding the conjugate solution to the wells. For color development, ABTS substrate was added and incubated for 30 min. BioTek Epoch Microplate Spectrophotometer (BioTek Instruments Inc., Winooski, VT, USA) was used to collect absorption values and recorded using Gen5 1.10 software.

#### 2.3.10. Statistical Analysis

All results were obtained at least thrice unless otherwise stated, to enable the presentation of the results as mean ± SD. Where necessary, two statistically different data were determined using the two-tailed Student’s *t*-test.

## 3. Results

### 3.1. Characterization of Nanoparticles

**Figure 1:**
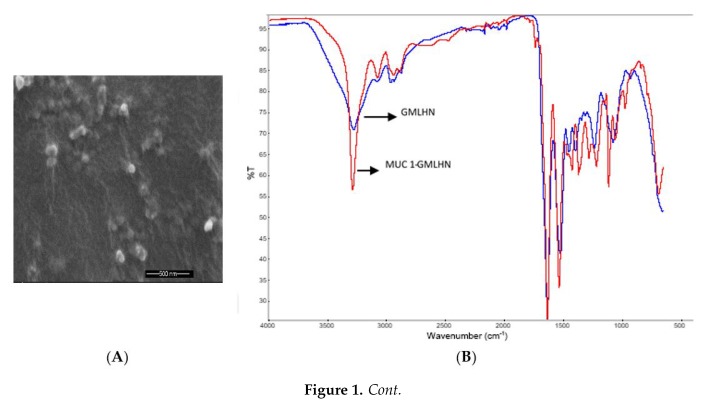

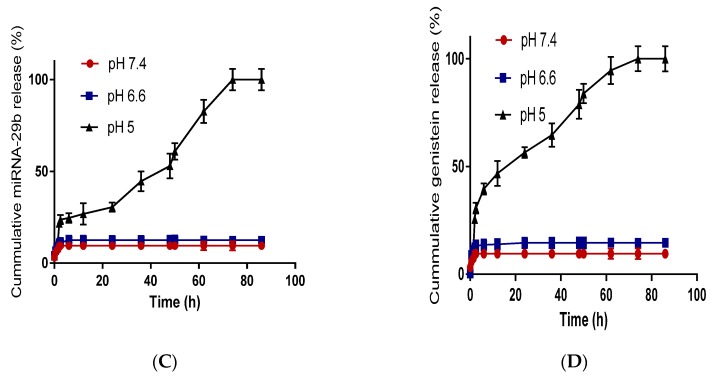
Nanoparticle characterization. (**A**) SEM microscopy of GMLHN showing the nanoparticles are spherical. (**B**) FT-IR spectra of GMLHN and MUC 1-aptamer confirming the covalent conjugation of aptamer to the nanoparticles. (**C**) Release profile of miRNA-29b from MUC 1-aptamer functionalized GMLHN at different pH values (**D**) Release profile of genistein from MUC 1-aptamer functionalized GMLHN at different pH values.

Figure 1A reveals that MUC 1-aptamer functionalized GMLHN nanoparticles are spherical in nature as captured by SEM. Particle size analysis by dynamic light scattering (Table 1) confirms that the GMLHN nanoparticles were bigger in size than the unfunctionalized GMLHN nanoparticles. The presence of MUC 1-aptamer on the surface of GMLHN nanoparticles led to the conversion of the surface charge from −2.2 mV from the unfunctionalized nanoparticles to 4.2 mV.

FT-IR spectra in Figure 1B demonstrate the differences in the spectra of both MUC 1-aptamer functionalized GMLHN and unfunctionalized GMLHN. FT-IR spectra in Figure 1B demonstrates the conjugation of anti-MUC 1 aptamer to GMLHN. The peak at 3274 cm^−1^ found in the non-functionalized GMLHN spectrum can be attributed to the amide stretching vibration of both NH_2_ and OH in the nanoparticles [13]. Interestingly, a more prominent and sharper peak was observed at the same wavelength probably due to the presence of additional NH_2_ and OH stretching vibration from the amide bond due to the conjugation of NH_2_ in the anti-MUC1 aptamer to the GMLHN. The peak at approximately 1541 cm^−1^, seen for both GMLHN and anti-MUC 1 aptamer functionalized GMLHN could be attributable amide II carbonyl stretching from human IgG [13]. Further, at approximately 1720 cm^−1^, a peak is present in the spectra for MUC 1-aptamer functionalized GMLHN but absent in the non-functionalized GMLHN. This peak is attributed to conjugated amide stretching due to the conjugation of NH_2_ from the aptamer to the carboxyl groups present in the GMLHN. Further, the NH (out of plane) bond at approximately 1112.74 cm^−1^ present in the aptamer functionalized GMLHN spectrum is attributable to the presence of the aptamer on the nanoparticles.

Release of genistein and miRNA-29b from MUC 1-aptamer functionalized GMLHN was monitored for a period of 90 h (approximately 4 days) at pH 5, 6.6 and 7.4 in Figure 1C,D. Optimal release was observed at pH value of 5, where 100% release of miRNA-29b and genistein was achieved before 80 h. However, less amount of both payloads were released at less acidic pH values of 6.6 and 7.4.

The EE of miRNA-29b and genistein in GMLHN were calculated to be 98.8 ± 0.4% and 99.2 ± 0.6% respectively while LC were calculated to be 8.6 ± 0.1% and 51.8 ± 0.5% respectively

### 3.2. Cellular Uptake of MUC 1-Aptamer Functionalized GMLHN

**Figure 2:**
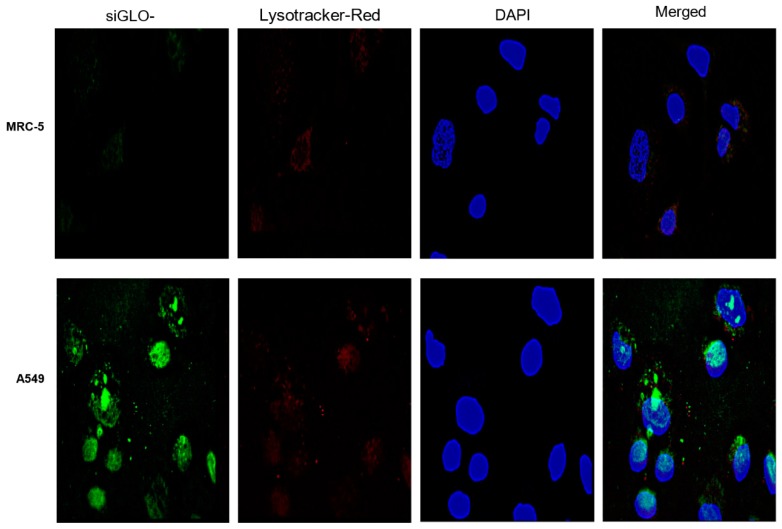
Intracellular delivery of siGLO-FAM loaded into MUC 1-aptamer functionalized nanoparticles. Upper panels show the nanoparticles in MRC-5 fibroblast cells while the lower panels show the delivery of nanoparticles to MUC 1 expressing A549 cells.

Fluorescence microscopy in Figure 2 shows limited delivery of MUC 1-aptamer functionalized nanoparticles to normal lung fibroblast MRC-5 cells. Conversely, adenocarcinoma A549 cells show a greater accumulation of MUC 1-aptamer functionalized nanoparticles intracellularly. Lysotracker-red was used to label late endosome/lysosomes while DAPI (blue) was used for labelling of the nucleus. The merged panel for both A549 especially, shows some co-localization of nanoparticles and endosome suggesting some nanoparticles were still in the endosome after 4 h of incubation.

### 3.3. Western Blot Analysis

**Figure 3:**
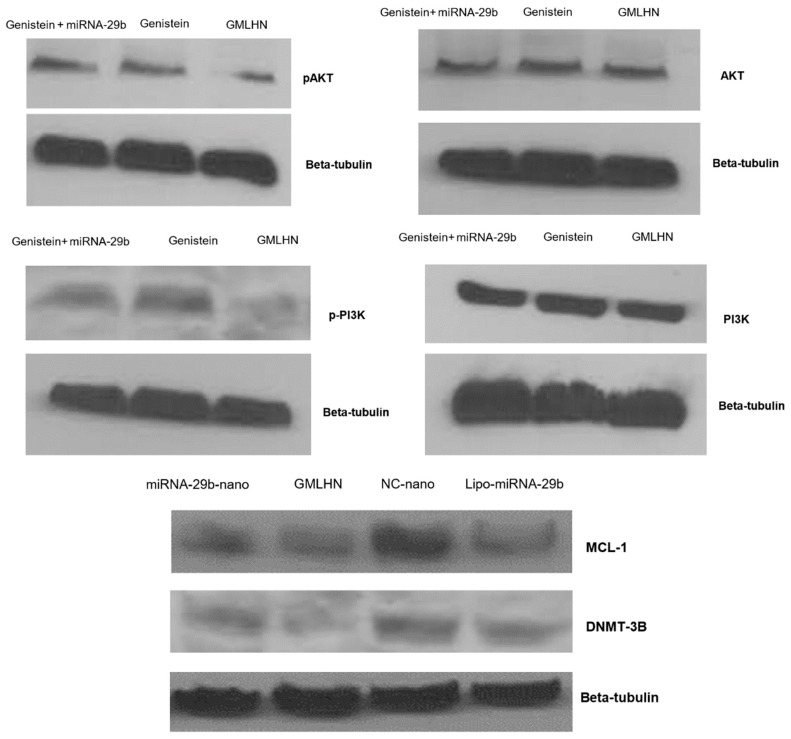
Downregulation of oncogenes in A549 cells. Western blot analysis was used to probe the effect of MUC 1-aptamer functionalized GMLHN on the expression of A. pAKT, B. AKT, C. p-PI3K, D. PI3K and E. both MCL-1 and DNMT-2B. NC-nano represents negative control miRNA-loaded hybrid nanoparticles, lipo-miRNA-29b represents lipofectamine-transfected miRNA-29b.

The effect of the combination of genistein and miRNA-29b loaded in the MUC 1-aptamer functionalized GMLHN nanoparticles on the expression of relevant oncoproteins was assessed using western blot analysis. Figure 3 demonstrates an efficient downregulation of pAKT by GMLHN in comparison to miRNA-29b-loaded nanoparticles as well as genistein powder. Similar trend was observed with p-PI3K, suggesting that GMLHN nanoparticles were able to stably inhibit the expression of these two oncoproteins in A549. In contrast, AKT and PI3K were not downregulated in A549 cells by any of the formulations, showing the specificity of genistein, whether loaded into a nanoparticle or used as a powder. Oncoproteins that are well known to be downregulated by miRNA-29b MCL-1 and DNMT3B were effectively downregulated by GMLHN nanoparticles even more efficiently than lipofectamine-200.

### 3.4. Antiproliferation Effect of GMLHN in A549 Cells

**Figure 4:**
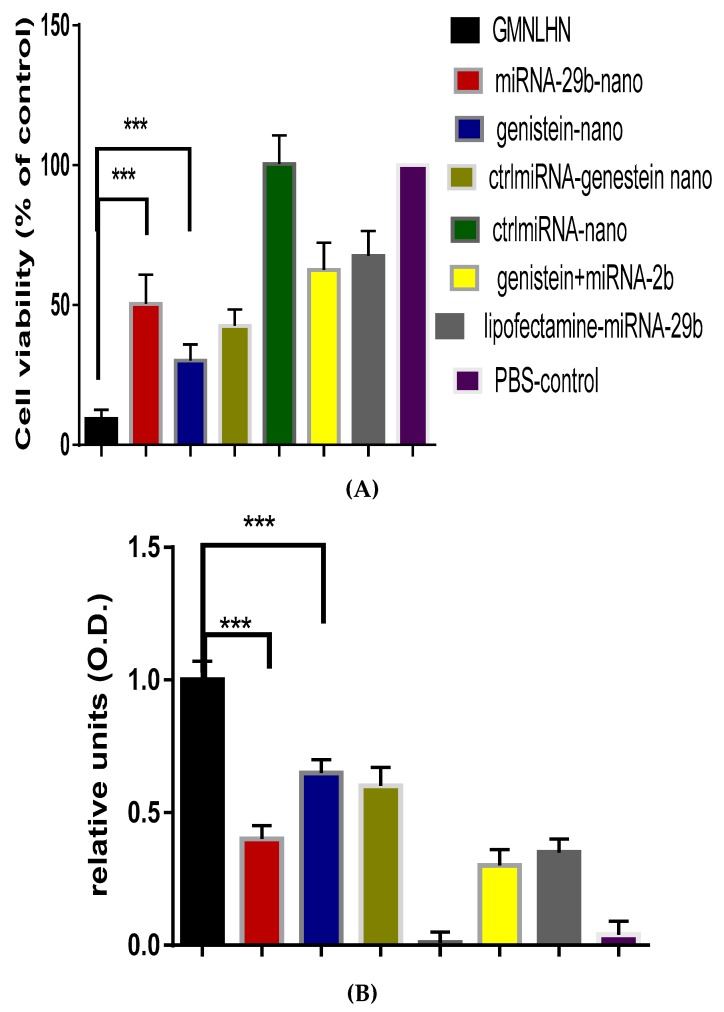
Antiproliferative effect and apoptosis. (**A**) MTT assay showing the antiproliferative effect of GMLHN, (**B**) Initiation of apoptosis in A549 cells as measured by cell death detection ELISA. *p* ≤ 0.001, *n* = 3. miRNA-29b-nano = miRNA-29b-loaded nanoparticles; genistein-nano = genistein-loaded nanoparticles; ctrlmiRNA-nano = controlmiRNA-loaded nanoparticles; genistein+miRNA-29b = physical mixture of genistein and miRNA-29b.

The antiproliferative effect of GMLHN nanoparticles on A549 cells was assessed by MTT assay and compared to various controls. Figure 4A demonstrates that GMLHN produced a far superior antiproliferative effect against A549 cells when compared to individual miRNA-29b and genistein-loaded nanoparticles. Similarly, in Figure 4B, GMLHN produced a superior apoptotic effect in A549 cells, as measured by cell-death detection ELISA, than all other formulations tested, confirming that nanoparticles loaded with a combination of miRNA-29b and genistein are more effective in inhibiting the growth of lung cancer cells than nanoparticles loaded with individual miRNA-29 and genistein.

## 4. Discussion

This study aimed to co-encapsulate miRNA-29b with genistein in our nanoparticles (GMLHN) to produce a combination therapy that will effectively treat NSCLC. The overarching hypothesis of this proposal is that MUC1-aptamer functionalized hybrid nanoparticle will efficiently deliver a combination of miRNA-29b and genistein to NSCLC. Successful delivery of genistein-miRNA-29b-loaded hybrid nanoparticles (GMLHN) will significantly inhibit NSCLC tumor by downregulating essential oncoproteins, phosphorylated AKT (pAKT), phosphorylated phosphoinositide-3 kinase (p-PI3K), myeloid cell leukemia sequence 1 (MCL 1) and DNA methyltransferase 3B (DNMT3B). miRNA-29b is known to regulate cell proliferation, differentiation and apoptosis, hence they have a significant role to play in cancer [1,14,15,16,17,18]. Expression of miRNA-29b is known to be reduced in lung cancer and other types of cancer [16]. Conversely, DNMTB expression was significantly reduced as a result of forced expression of miRNA-29b by direct interaction at both protein and mRNA levels [1]. Further, MCL1, a potent oncoproteins which belongs to the BCL-2 family is known to be inhibited miR-29b in cancer cells [1].

Genistein is a natural isoflavonoid that is abundantly found in soy products. It increases the anti-neoplastic effect of some chemotherapeutic agents, hence is a potential drug in multiple tumor types [7]. Previously, genistein has been reported to inhibit the growth of lung cancer by downregulating essential oncoproteins, phosphorylated AKT (pAKT), and phosphorylated phosphoinositide-3 kinase (p-PI3K) (7). pAKT is an important factor in the growth and survival of tumour cells often enabling the chemoresistance of cancer cells. Chemoresistance of cancer cells has been shown severally to be significantly regulated by the PI3K/AKT pathway [18,19]. AKT phosphorylation has been shown to play a role as a prognostic tool in breast, prostate and non-small cell lung cancer [20,21].

Considering the fact that miRNA-29b and genistein attack different targets in cancer, we hypothesize that the combination of miRNA-29b and genistein will provide an additive effect in the treatment of NSCLC. However, to ensure precise and targeted delivery, these molecules were loaded into hybrid nanoparticles fabricated with a combination of human IgG and poloxamer-188. These nanoparticles were than functionalized with MUC 1-aptamer to enable a selective delivery of these payloads to NSCLC. These hybrid nanoparticles functionalized with MUC 1-aptamer, loaded with miRNA-29b as a single therapy have been previously characterized extensively to ensure consistent production [22,23]. We were also able to show that MUC 1 protein is expressed in NSCLC but not in normal healthy cells [21].

In this present study, we loaded both miRNA-29b and genistein in these nanoparticles to elicit an additive effect in the treatment of NSCLC.

As shown in Figure 1A, the spontaneous production of the nanoparticles lead to the production of spherical particles. FT-IR spectroscopy was used to confirm the covalent conjugation of anti-MUC 1 aptamer to the nanoparticles. Detailed explanation of crucial peaks in the FT-IR spectra was given in the result section. In Figure 1C,D, miRNA-29b and genistein were efficiently released at pH 5 unlike at pH 7.4 and 6.6. The significance of this is that pH 5 represents the pH value for late endosome/lysosome, while pH values 7.4 and 6.6 represent the approximate pH values for blood and tumor microenvironment respectively [24,25]. Ideally, we do not expect the payloads to be released in the blood or in the tumor microenvironment but in the cytoplasm of cancer cells. The acidic pH of the endosome facilitates the gradual dissolution of the IgG in the endosome which allows a steady release of the payload in the endosome. This is because the pH of the endosome is lower than the isoelectric point of human IgG which is 7. Hence, enabling the gradual dissolution of the nanoparticles at pH 5. miRNA-29b and genistein had limited released at pH values 6.6 possibly due to the fact that IgG is not soluble at pH values very close the its isoelectric point. Same argument could be made for the low release at pH 7.4. Proteins typically do not dissolve at pH values close to their isoelectric point [12]. This concept is backed by the efficient internalization of siGLO-FAM in Figure 2, which demonstrates the successful delivery of the loaded siGLO-FAM in the cytoplasm of A549 cells, enabled by the presence of MUC 1 on the surface of these cells. In contrast, siGLO-FAM was not efficiently delivered to the cytoplasm of MRC-5 due to the absence of MUC 1 on the surface of MRC-5 cells. The increase in the diameter of the MUC 1-aptamer functionalized GMLHN nanoparticles over the nonfunctionalized counterpart is mainly due to the presence of aptamer on the surface of the nanoparticles.

The negative charge on the nonfunctionalized GMLHN nanoparticles could be attributable to the presence of residual miRNA on the surface of the nanoparticles. In contrast, the positive charge on the MUC 1-aptamer functionalized nanoparticles could be attributed to the amino group (NH2) in the aptamer. The presence of hycrochloric acid lead to the loss of the lone pair of electrons on the amino group resulted in the positive charges.

The main objective behind our codelivery of miRNA-29b and genistein is to effect the simultaneous induction of cell death by an anticancer drug (genistein) and suppression of antiapoptotic defense/stimulation of apoptosis (miRNA-29b) to enhance the treatment of NSCLC. miRNA-29b is known to limit cancer growth by regulating the proliferation of cells and enhancing apoptosis [21]. MiRNA-29b is known to downregulate DNMT3B and MCL1 amongst other oncogenes [6,26]. Genistein, on the other hand, inhibits lung cancer cell growth by downregulating essential oncoproteins, pAKT) and p-PI3K (7). Figure 3 demonstrates that GMLHN nanoparticles were able to downregulate both pAKT and p-PI3K in A549 cells, better in comparison to miRNA-29b-loaded nanoparticles and unencapsulated genistein. Although, both oncoproteins were downregulated, the total levels of PI3K or AKT were not altered, which are needed for normal cell function. Similarly, oncoproteins DNMT3B and MCL1, were efficiently downregulated by anti-MUC 1 aptamer functionalized GMLHN nanoparticles than miRNA-29b-loaded nanoparticles, lipofectamine-miRNA-29b, negative control and miRNA-loaded nanoparticles. These results suggest that the MUC 1-functioanlized GMLHN nanoparticles were very effective in downregulating the targeted oncoproteins. The effect of the downregulation of these oncoproteins on the anti-cancer effect of GMLHN is demonstrated in Figure 4A, which shows that GMLHN demonstrated a more potent growth inhibition in A549 cells when compared to other formulations tested. Further, reduced expression of these oncoproteins led to increased apoptosis in A549 cells, more effectively that individually loaded miRNA-29b and genistein in the nanoparticles. The amount of miRNA-29b and genistein used for nanoparticle production was based on the ratio we plan to use for in vivo study in mice. Our previous study demonstrated that our nanoparticles containing up to 20 mg/mL of miRNA-29b are well tolerated and previous report had shown that miRNA-29b is most effective in NSCLC tumor-bearing mice at a dose of 1.5 mg/kg [16]. Further, genistein loaded in our nanoparticle was found to be tolerated by mice up to a dose of 10 mg/kg, hence we decided to use a of dose 9 mg/kg in future mice studies. The ratio of the doses for both miRNA-29b and genistein resulted in 1:6 (miRNA-29b:genistein), hence, nanoparticles were fabricated with 0.73 mg and 4.38 mg of miRNA-29b and genistein (1:6) respectively. The LC of both miRNA-29b and genistein in GMLHN reflected a 1:6 ratio (8.6:51.1%). These cells were treated with GMLHN containing an equivalent of 50 nM of miRNA-29 and 1.6 M of genistein. These concentrations are equivalent to 1:6 ratio by weight of miRNA-29b and genistein. The same concentrations of 50 nM and 1.6 M of miRNA-29b and genistein was used for all the relevant formulations.

## 5. Conclusions

Results generated were able to demonstrate that genistein-miRNA-29b-loaded hybrid nanoparticles (GMLHN) could be a potential treatment modality for NSCLC because of the ability of the payloads to attack multiple targets, hence resulting in an increased anti-cancer effect. Further, increased apoptosis was demonstrated by GMLHN over individual miRNA-29b and genistein-loaded nanoparticles.

## Figures and Tables

**Table 1:** Particle size analysis by dynamic light scattering and net charge on nanoparticles.

Samples	Particle Size ± SD (nm)	PDI * ± SD	Zeta Potential ± SD (ζ, mV)
GMLHN	240.8 ± 41.2	0.242 ± 0.021	−2.2 ± 1.8
MUC 1-aptamer functionalized GMLHN	598 ± 34.1	0.414 ± 0.241	4.2 ± 1.2

* PDI: Polydispersity Index.
